# Lumpy skin disease virus protein LSDV122 impairs IFN-I receptor complex formation to evade host innate immunity

**DOI:** 10.1371/journal.ppat.1013871

**Published:** 2026-01-12

**Authors:** Meng-Yao Sun, Li-Bo Cao, Qin-Ling Wan, Zhen-Zhen Li, Jun-Zheng Du, Hong-Bing Shu, Yu-Lin Yang

**Affiliations:** 1 State Key Laboratory of Animal Disease Control and Prevention, College of Veterinary Medicine, Lanzhou University, Lanzhou Veterinary Research Institute, Chinese Academy of Agricultural Sciences, Lanzhou, China; 2 Gansu Province Research Center for Basic Disciplines of Pathogen Biology, Lanzhou Veterinary Research Institute, Chinese Academy of Agricultural Sciences, Lanzhou, China; 3 Department of Infectious Diseases, Medical Research Institute, Taikang Center for Life and Medical Sciences, Zhongnan Hospital of Wuhan University, Wuhan University, Wuhan, China; Thomas Jefferson University, UNITED STATES OF AMERICA

## Abstract

Lumpy skin disease virus (LSDV) is a pathogenic poxvirus that causes systemic disease in cattle. Although LSDV encodes multiple proteins that are predicted to regulate host defense, the underlying mechanisms of its immune evasion strategies remain largely elusive. Here we identify the LSDV-encoded protein LSDV122 as an antagonist of type I interferon (IFN-I)-mediated innate immunity. LSDV122 interacts with both subunits of the IFN-I receptor, IFNAR1 and IFNAR2, disrupting their proper assembly and preventing the recruitment of the downstream kinases JAK1 and TYK2, leading to impairment of IFN-β-mediated JAK-STAT signaling and induction of antiviral IFN-stimulated genes (ISGs). Deletion of the *LSDV122* gene (LSDVΔ122) led to stronger antiviral response by restoring IFN-β-induced signaling *in vitro* and in a mouse model. Our study suggests that LSDV122 plays a critical role in antagonizing IFN-I signaling and is essential for efficient viral immune evasion, offering new insights into the rational design of live-attenuated LSDV vaccines.

## Author summary

Lumpy skin disease virus (LSDV) causes severe disease in cattle and poses a growing threat to global livestock health and production. How LSDV evades the host defense remains enigmatic. In this study, we identify LSDV122 as a viral immunomodulatory protein that specifically antagonizes IFN-β-triggered JAK-STAT signaling by impairing the formation of the IFNAR-JAK signaling complex. Deletion of LSDV122 enhances antiviral response both *in vitro* and *in vivo*, highlighting its essential role in immune evasion. These findings may guide the development of attenuated vaccines or novel antiviral strategies targeting immune modulators encoded by LSDV.

## Introduction

Lumpy skin disease virus (LSDV), a member of the genus Capripoxvirus in the family Poxviridae, is the etiological agent of lumpy skin disease (LSD), an emerging vector-borne disease of cattle with substantial economic impacts on the livestock industry [1,2]. Clinically, affected cattle exhibit symptoms of fever, lacrimation, anorexia, and lymphadenopathy, followed by firm skin nodules that initially appear on the head, neck, and abdomen and may eventually disseminate over the entire body surface [3,4]. Since its first identification in Zambia in 1929, LSD has rapidly expanded its geographic range in recent decades and is currently listed as a notifiable disease by the World Organisation for Animal Health (WOAH) [5–7]. However, progress in developing preventive and control strategies is hampered by the limited understanding of LSDV infection, pathogenesis, and immune evasion mechanisms [8,9].

Type I interferons (IFNs), particularly IFN-β, play a central role in host antiviral defense by triggering the transcription of interferon-stimulated genes (ISGs) [10]. Upon recognition of invading viruses by pattern recognition receptors (PRRs), IFN-β is rapidly produced as part of the innate immune response. IFN-β exerts its antiviral effects by binding to the heterodimeric receptor complex IFNAR, which consists of IFNAR1 and IFNAR2 subunits. This ligand-receptor engagement activates Janus kinase 1 (JAK1) and tyrosine kinase 2 (TYK2), which are constitutively associated with IFNAR2 and IFNAR1 respectively [11,12]. Subsequently, the transcription factors STAT1 and STAT2 are phosphorylated by JAK1 and, together with IFN regulatory factor 9 (IRF9), form the interferon-stimulated gene factor 3 (ISGF3) complex. The ISGF3 complex translocates to the nucleus and specifically binds to IFN-stimulated response elements (ISREs), thereby inducing the transcription of downstream ISGs [13,14]. Proteins encoded by most ISGs contribute to antiviral defense through various mechanisms, including inhibition of virus entry, replication, translation, assembly and release [10,15–17].

Several poxviruses have evolved diverse strategies to antagonize IFN-I response, such as producing soluble IFN-binding proteins and expressing intracellular proteins that disrupt JAK/STAT signaling or block ISGF3 formation [18–21]. Although these mechanisms are well characterized in other poxviruses, how LSDV interferes with IFN-I signaling has not been elucidated. In this study, we identified the LSDV-encoded protein LSDV122 as a negative regulator of IFN-β-mediated innate immune response. Our results indicated that LSDV122 suppressed IFN-β-triggered JAK-STAT signaling by interacting with both IFNAR1 and IFNAR2, thereby disrupting the assembly of the receptor complex and preventing the recruitment of the downstream kinases JAK1 and TYK2. The LSDV122-deficient virus (LSDVΔ122) exhibited an enhanced ability to induce antiviral response by restoring IFN-β-induced signaling *in vitro* and enhancing virus-induced immune response in a mouse model. Our findings reveal a novel immune evasion mechanism employed by LSDV and highlight the role of LSDV122 in subverting host IFN-I response, which may help for rational design of live-attenuated LSDV vaccines.

## Results

### LSDV122 specifically inhibits IFN-β-triggered JAK-STAT signaling

**Fig 1:**
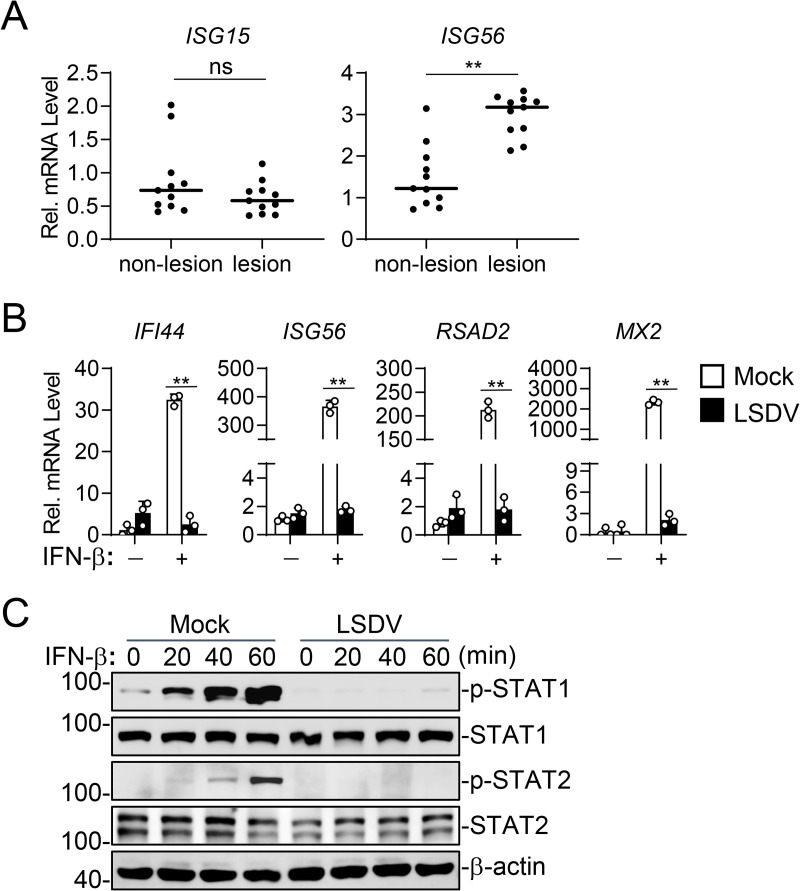
LSDV inhibits IFN-β-triggered JAK-STAT signaling. A. The mRNA levels of *ISG15* and *ISG56* genes on lesion and adjacent non-lesion skin samples *in vivo*. Holstein cattle were intravenously injected with LSDV for 14 days, and then lesion and non-lesion skin samples from the same cattle were dissected for RT-qPCR analysis of mRNA levels of *ISG15* and *ISG56* genes. B. Effects of LSDV on IFN-β-induced transcription of ISGs. MDBK cells (5 × 10^5^) were left un-infected or infected with wild-type LSDV (MOI = 1) for 12 hours and then treated with IFN-β (100 ng/ml, final concentration) for 6 hours followed by RT-qPCR analysis of mRNA levels of the indicated genes. C. Effects of LSDV on IFN-β-induced phosphorylation of STAT1 and STAT2. MDBK (5 × 10^5^) cells were left uninfected or infected with wild-type LSDV (MOI = 1) for 12 hours and then treated with IFN-β (100 ng/ml) for the indicated times before immunoblotting analysis with the indicated antibodies. Data shown in A-B are mean ± SD (n = 11 in A and n = 3 in B) from one representative experiment. These experiments were repeated at least twice with similar results. ns nonsignificant, **P < 0.01 (unpaired t-test).

Poxviruses have evolved diverse strategies to antagonize IFN-I response. To investigate whether LSDV employs similar mechanisms, we firstly examined the magnitude of IFN-I response during LSDV infection. We established a LSDV infection model in Holstein cattle and performed RT-qPCR on lesion and adjacent non-lesion skin samples. The results showed that the ISG15 mRNA levels in non-lesion and lesion samples were comparable without significant difference, while the ISG56 mRNA levels in lesion samples were only ~2-fold increase than the non-lesion samples (Fig 1A). These results suggest that the lesion samples from LSDV-infected cattle have a very limited activation of IFN-I response. Similarly, in Madin-Darby bovine kidney (MDBK) cells, a well-characterized bovine epithelial cell line commonly used as a natural host model for LSDV, we observed that LSDV infection failed to effectively induce transcription of ISGs including *IFI44*, *ISG56*, *RSAD2* and *MX2* genes and significantly suppressed IFN-β-induced transcription of these ISGs (Fig 1B). Consistently, immunoblotting analysis showed that LSDV infection markedly inhibited IFN-β-induced phosphorylation of STAT1 and STAT2, key transcription factors in the IFN-β signaling pathway (Fig 1C). These findings suggest that LSDV inhibits IFN-β-triggered antiviral response.

**Fig 2:**
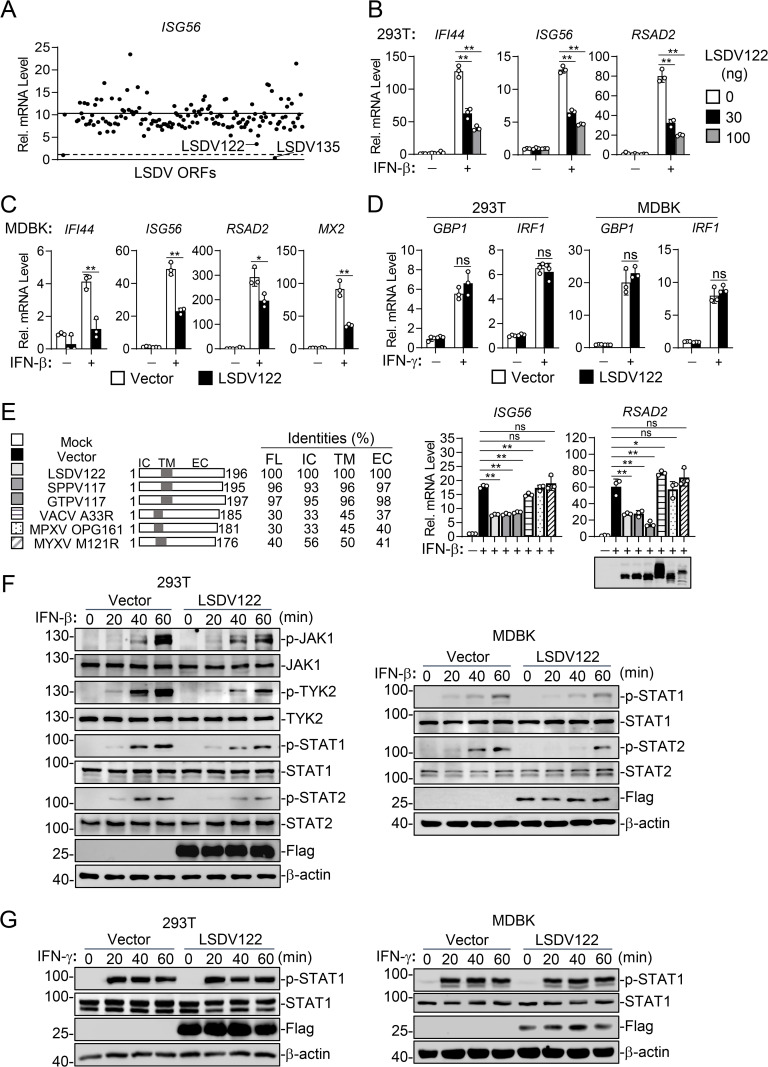
LSDV122 inhibits IFN-β-triggered JAK-STAT signaling. A. Screening of LSDV proteins that regulate IFN-β-induced signaling. HEK293T cells (2 × 10^5^) were transfected with an empty vector or LSDV mammalian expression plasmids for 24 hours. The cells were then left untreated or treated with IFN-β for 10 hours before RT-qPCR experiments to measure *ISG56* mRNA levels. All data were normalized to empty vector-transfected cells (dashed line). IFN-β-induced *ISG56* mRNA level in empty vector-transfected cells was indicated by the solid line. B. Effects of LSDV122 on IFN-β-induced transcription of downstream genes in HEK293T cells. HEK293T cells (2 × 10^5^) were transfected with either an empty vector or increased amounts of LSDV122-expressing plasmid for 24 hours. The cells were then left untreated or treated with IFN-β (100 ng/ml) for 10 hours before RT-qPCR experiments to measure mRNA levels of the indicated genes. C. Effects of LSDV122 on IFN-β-induced transcription of downstream genes in MDBK cells. MDBK cells were transduced with either an empty vector or LSDV122-expressing plasmid to establish stable cell lines. The cells (2 × 10^5^) were left untreated or treated with IFN-β (100 ng/ml) for 6 hours before RT-qPCR experiments. D. Effects of LSDV122 on IFN-γ-induced transcription of downstream genes. HEK293T cells (2 × 10^5^) transfected with either an empty vector or LSDV122-expressing plasmid for 24 hours, or LSDV122-expressing and control MDBK cells (2 × 10^5^) were left untreated or treated with IFN-γ (100 ng/ml) for 10 hours before RT-qPCR experiments to measure the mRNA levels of *GBP1* and *IRF1* genes. E. Effects of LSDV122 homologous proteins on IFN-β-induced transcription of downstream genes. HEK293T cells (2 × 10^5^) were transfected with the indicated plasmids for 24 hours. The cells were then left untreated or treated with IFN-β (100 ng/ml) for 10 hours before RT-qPCR experiments to measure the mRNA levels of *ISG56* and *RSAD2* genes. Sequence identities of LSDV122 among orthopoxviruses are shown below. Full length (FL), Intracellular (IC), Transmembrane (TM) and Extracellular (EC). F. Effects of LSDV122 on IFN-β-induced phosphorylation of downstream signaling components. HEK293T cells (2 × 10^5^) transfected with either an empty vector or LSDV122-expressing plasmid for 24 hours, or LSDV122-expressing and control MDBK cells (2 × 10^5^) were left untreated or treated with IFN-β (100 ng/ml) for the indicated times before immunoblotting analysis with the indicated antibodies. G. Effects of LSDV122 on IFN-γ-induced phosphorylation of STAT1. HEK293T cells (2 × 10^5^) transfected with either an empty vector or LSDV122-expressing plasmid for 24 hours, or LSDV122-expressing and control MDBK cells (2 × 10^5^) were left untreated or treated with IFN-γ (100 ng/ml) for the indicated times before immunoblotting analysis with the indicated antibodies. Data shown in B-D are mean ± SD (n = 3) from one representative experiment. These experiments were repeated at least twice with similar results. ns nonsignificant, *P < 0.05, **P < 0.01 (unpaired t-test).

To identify potential viral inhibitors of IFN-β signaling, we performed a genome-wide screen of LSDV-encoded proteins for their abilities to regulate IFN-β-induced transcription of *ISG56* gene by RT-qPCR experiments in HEK293T cells, a human embryonic kidney cell line with high transfection efficiency and fully IFN-β response [22,23]. These screens indicated that LSDV-encoded proteins LSDV122 and LSDV135 markedly inhibited IFN-β-induced transcription of *ISG56* in HEK293T cells (Fig 2A). Given that LSDV135 is predicted to be a homolog of the vaccinia virus B18R protein, a known soluble IFN-I decoy receptor [19,24,25], we focused our investigation on LSDV122 in this study, whose functions remain poorly characterized. Further experiments demonstrated that ectopic expression of LSDV122 inhibited IFN-β-induced transcription of *IFI44*, *ISG56* and *RSAD2* genes in a dose-dependent manner in HEK293T cells (Fig 2B). To confirm the functions of LSDV122 in the natural host cells of LSDV, we established a MDBK cell line stably expressing LSDV122 through lentiviral transduction. Consistently, ectopic expression of LSDV122 inhibited IFN-β-induced transcription of *IFI44*, *ISG56*, *RSAD2* and *MX2* genes in MDBK cells (Fig 2C). In similar experiments, LSDV122 had no marked effects on IFN-γ-induced transcription of *GBP1* and *IRF1* genes in HEK293T and MDBK cells (Fig 2D), suggesting that LSDV122 specifically antagonizes IFN-I but not IFN-II signaling. We further evaluated the functions of LSDV122 homologs encoded by related poxviruses. Homologous proteins from closely related Capripoxviruses, including sheeppox virus (SPPV) and goatpox virus (GTPV), which share >95% amino acid sequence identity with LSDV122, inhibited IFN-β-induced transcription of *ISG56* and *RSAD2* genes. However, LSDV122 homologs from vaccinia virus (VACV), monkeypox virus (MPXV), and myxoma virus (MYXV), which share <50% amino acid sequence identity, had little effect on IFN-β-induced transcription of these ISG genes, which likely reflects limited amino acid conservation or host-specific differences (Fig 2E). Biochemically, ectopic expression of LSDV122 inhibited IFN-β-induced phosphorylation of JAK1, TYK2, STAT1 and STAT2 in HEK293T cells, and phosphorylation of STAT1 and STAT2 in MDBK cells, events that represent hallmarks of IFN-I signaling (Fig 2F). In contrast, LSDV122 had no marked effects on IFN-γ-induced phosphorylation of STAT1 in HEK293T and MDBK cells (Fig 2G). Collectively, these results suggest that LSDV122 specifically inhibits IFN-β-triggered JAK-STAT signaling.

### LSDV122 interacts with IFNAR1 and IFNAR2

**Fig 3:**
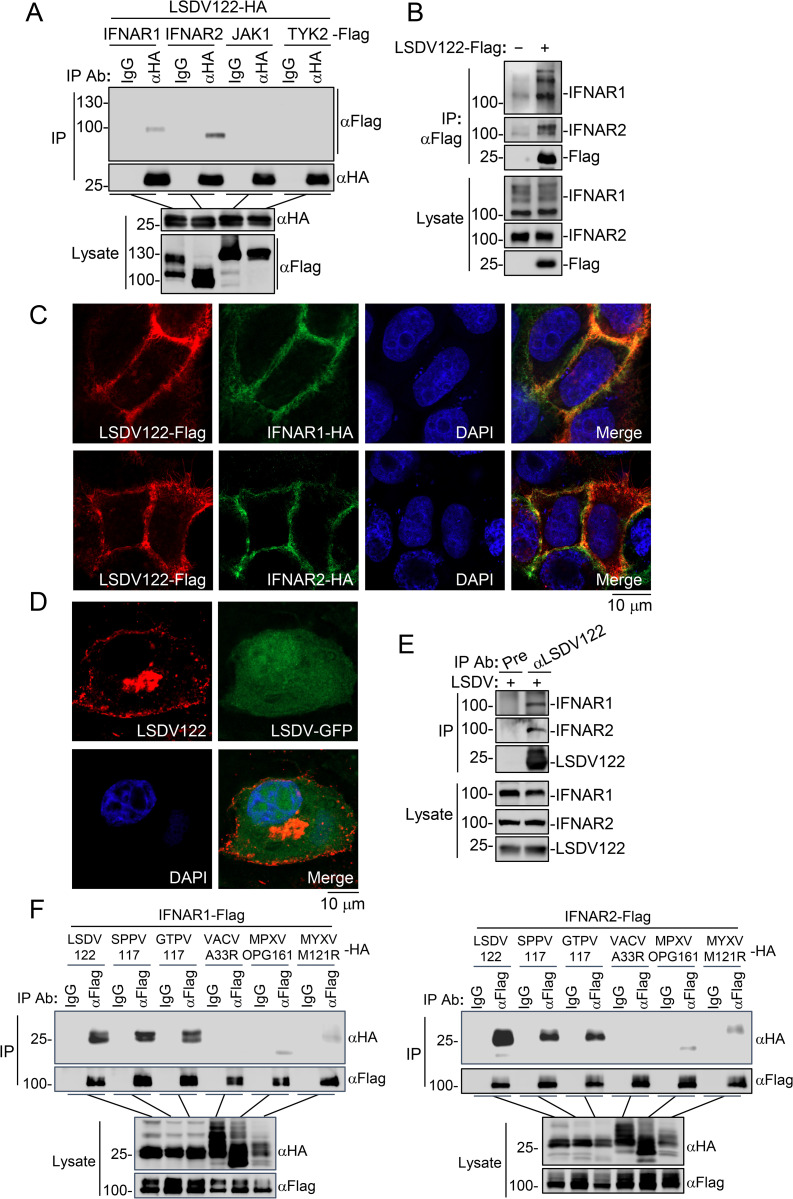
LSDV122 interacts with IFNAR1 and IFNAR2. A. The interaction of LSDV122 with IFNAR1 and IFNAR2 in mammalian overexpression system. HEK293T cells (5 × 10^6^) were transfected with the indicated plasmids for 24 hours and then lysed for coimmunoprecipitation with control IgG or anti-HA antibody, followed by immunoblotting analysis with the indicated antibodies. B. The interaction of LSDV122 with endogenous IFNAR1 and IFNAR2. HEK293T cells (2 × 10^7^) were transfected with an empty vector or LSDV122-expressing plasmid for 24 hours and then lysed for coimmunoprecipitation with anti-Flag antibody, followed by immunoblotting analysis with the indicated antibodies. C. Co-localization of LSDV122 with IFNAR1 and IFNAR2. HeLa cells (2 × 10^4^) were transfected with the indicated plasmids for 20 hours and then fixed for immunostaining before subjected to confocal microscopy. D. Subcellular distribution of LSDV122 during LSDV infection. MDBK cells (6 × 10^4^) were infected with LSDV-GFP (MOI = 2) for 18 hours and then fixed for immunostaining before subjected to confocal microscopy. E. The interaction of endogenous LSDV122 with IFNAR1 and IFNAR2 during LSDV infection. MDBK cells (5 × 10^7^) were infected with LSDV (MOI = 2) for 18 hours and the cells were harvested for immunoprecipitation with pre-immune serum or anti-LSDV122 serum. The lysates and immunoprecipitates were subjected to immunoblot analysis with the indicated antibodies. F. The interactions of LSDV122 and its homologous proteins with IFNAR1 and IFNAR2 in mammalian overexpression system. HEK293T cells (5 × 10^6^) were transfected with the indicated plasmids for 24 hours and then lysed for coimmunoprecipitation with control IgG or anti-Flag antibody, followed by immunoblotting analysis with the indicated antibodies. These experiments were repeated at least twice with similar results.

Since our earlier experiments showed that LSDV122 inhibited IFN-β-induced phosphorylation of JAK1, TYK2 and its downstream components (Fig 2F), we hypothesized that LSDV122 functions at or upstream of the level of JAK1 and TYK2 in the IFN-β signaling pathway. To test this, we examined the association of LSDV122 with IFNAR subunits and their associated kinases. Transient transfection and co-immunoprecipitation experiments indicated that LSDV122 was associated with IFNAR1 and IFNAR2 but not with JAK1 or TYK2 (Fig 3A). Furthermore, we confirmed the interaction of ectopically expressed LSDV122 with endogenous IFNAR1 and IFNAR2 (Fig 3B). Confocal microscopy showed that LSDV122 was localized to the cell membrane and colocalized with IFNAR1 and IFNAR2 (Fig 3C). LSDV122 is predicted to be an extracellular enveloped virion (EEV) membrane protein based on its homology to VACV A33R [25]. We examined its intracellular distribution following LSDV infection by immunofluorescent microscopy. The results showed that LSDV-encoded LSDV122 was localized both in the cytoplasm and on the plasma membrane (Fig 3D). This distribution pattern is consistent with several well-characterized poxviral membrane proteins, such as VACV A33R, A36R, and B5R, which transit through host secretory and vesicular compartments and can reside on host cellular membranes at intermediate stages of infection before being incorporated into the EEV outer membrane during virion wrapping and egress [26–28]. We also examined the endogenous interactions of LSDV122 with IFNAR1 and IFNAR2 during LSDV infection. The results indicated that LSDV-encoded LSDV122 was associated with endogenous IFNAR1 and IFNAR2 following infection in MDBK cells (Fig 3E). Additionally, co-immunoprecipitation assays showed that LSDV122 and its Capripoxvirus orthologs SPPV117 and GTPV117 strongly interacted with IFNAR1 and IFNAR2, whereas VACV A33R, MPXV OPG161, and MYXV M121R exhibited only weak or negligible interactions, which is consistent with the distinct functions in interfering IFN-I signaling observed among these orthologues (Fig 3F). Collectively, these results suggest that LSDV122 targets IFNAR1 and IFNAR2.

**Fig 4:**
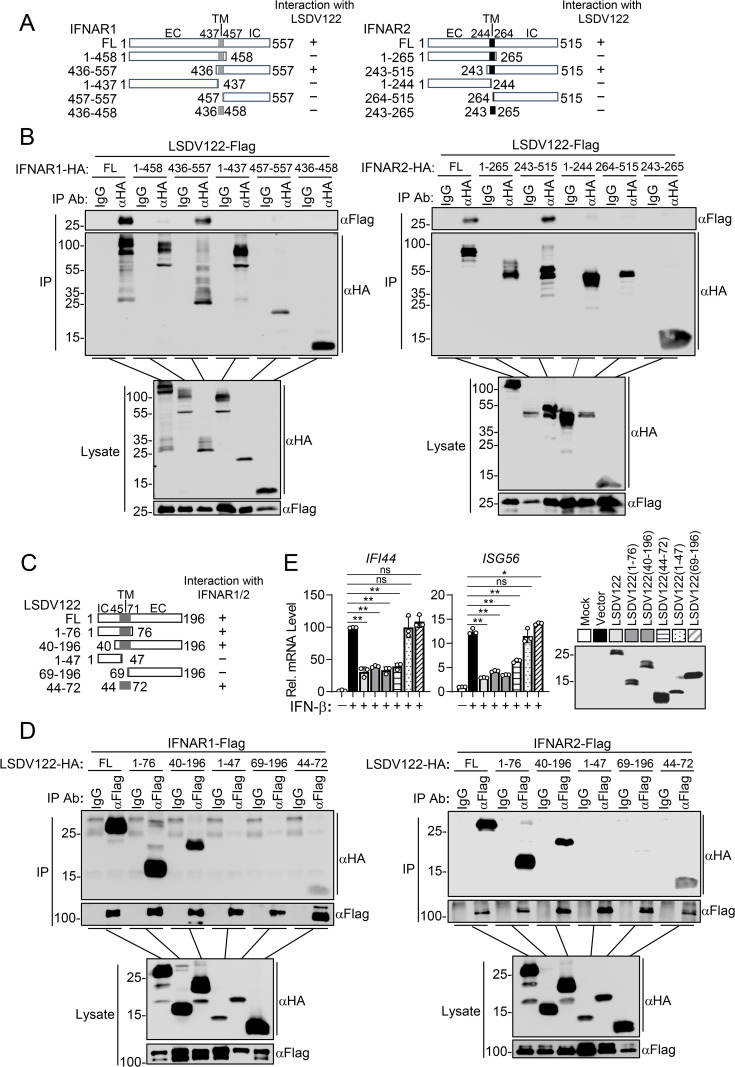
Domain mapping of the interactions between LSDV122 and IFNAR1 or IFNAR2. A. A schematic presentation of IFNAR1 and IFNAR2 truncations and their abilities to interact with LSDV122. − , no interaction; + , positive interaction. B. The interaction of IFNAR1 or IFNAR2 truncations with LSDV122. HEK293T cells (5 × 10^6^) were transfected with the indicated plasmids for 24 hours, followed by coimmunoprecipitation and immunoblotting analysis with the indicated antibodies. C. A schematic presentation of LSDV122 truncations and their abilities to interact with IFNAR1 and IFNAR2. D. The interaction of LSDV122 truncations with IFNAR1 or IFNAR2. HEK293T cells (5 × 10^6^) were transfected with the indicated plasmids for 24 hours, followed by coimmunoprecipitation and immunoblotting analysis with the indicated antibodies. E. Effects of LSDV122 and its truncations on IFN-β-induced transcription of downstream genes. HEK293T cells (2 × 10^5^) were transfected with either an empty vector, LSDV122 or its truncations for 20 hours. The cells were then left untreated or treated with IFN-β (100 ng/ml) for 10 hours before RT-qPCR experiments. Data shown are mean ± SD (n = 3) from one representative experiment. These experiments were repeated at least twice with similar results. ns nonsignificant, *P < 0.05, **P < 0.01 (unpaired t-test).

To delineate the specific regions of IFNAR1 and IFNAR2 responsible for interacting with LSDV122, we generated a series of truncation mutants (Fig 4A). Domain mapping experiments showed that LSDV122 specifically interacted with IFNAR1 and IFNAR2 deletion mutants containing both the transmembrane and cytoplasmic regions, but failed to interact with IFNAR1 and IFNAR2 mutants containing only the cytoplasmic fragment, transmembrane domain, extracellular fragment, or transmembrane and extracellular regions (Fig 4B). We next mapped the regions of LSDV122 responsible for its interactions with IFNAR1 and IFNAR2. LSDV122 is a 196-aa type II transmembrane protein, which contains a N-terminal cytoplasmic domain (aa 1–44), a middle transmembrane domain (aa 45–71), and a C-terminal extracellular domain (aa 72–196) (Fig 4C). Co-immunoprecipitation experiments indicated that LSDV122 mutants containing the transmembrane domain and cytoplasmic fragment strongly interacted with IFNAR1 and IFNAR2, while the transmembrane alone or the mutant containing transmembrane and extracellular domains interacted with IFNAR1 and IFNAR2 to a weaker degree. The LSDV122 mutants containing only the cytoplasmic fragment or the extracellular domain failed to interact with IFNAR1 and IFNAR2 (Fig 4D). These results suggest that the transmembrane domain of LSDV122 is essential for its interaction with IFNAR1 and IFNAR2. RT-qPCR analysis showed that the transmembrane domain of LSDV122 alone was sufficient to inhibit IFN-β-induced transcription of the *IFI44* and *ISG56* genes, while the extracellular domain or intracellular fragment alone had no such inhibitory effects (Fig 4E).

### LSDV122 disrupts IFNAR complex formation and kinase recruitments

**Fig 5:**
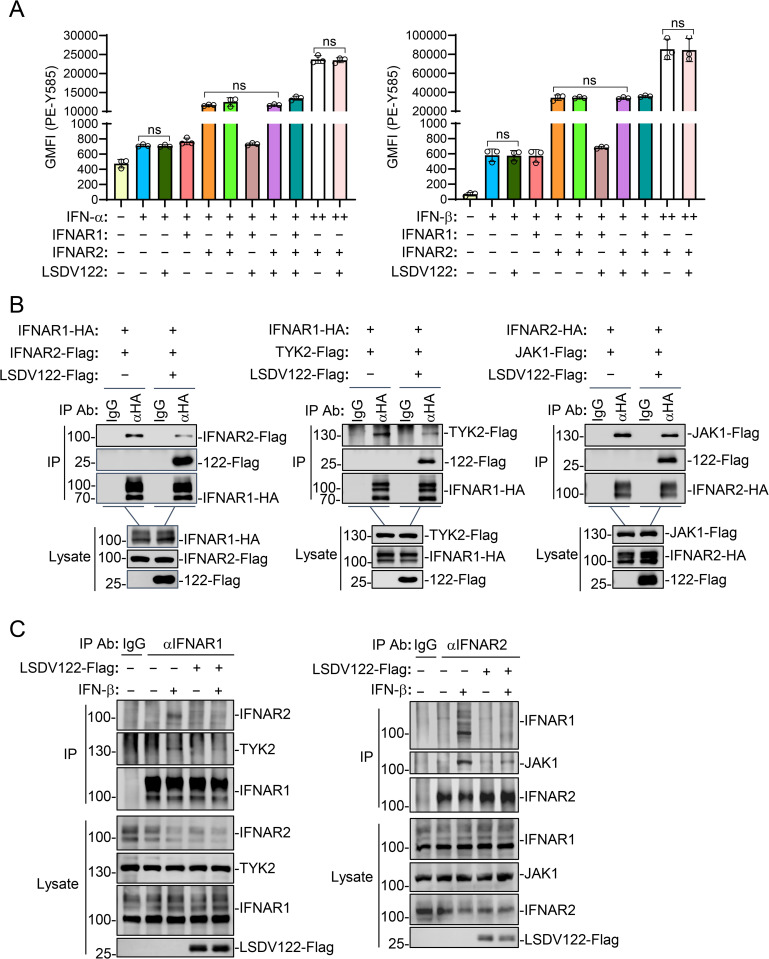
LSDV122 disrupts IFNAR complex formation and kinase recruitments. A. Effects of LSDV122 on binding of IFN-I to IFNAR. HEK293T cells (2 × 10^5^) were transfected with the indicated plasmids for 24 hours. The cells were then incubated with Flag-tagged IFN-α2 or IFN-β (+, 200 ng; ++, 800 ng) for 30 minutes at 4°C, followed by surface staining with an anti-Flag antibody. The binding of IFN-α2 or IFN-β to the cell surface was analyzed by flow cytometry. B. Effects of LSDV122 on IFNAR complex formation and its recruitment of downstream kinases. HEK293T cells (5 × 10^6^) were transfected with the indicated plasmids for 24 hours, followed by coimmunoprecipitation and immunoblotting analysis with the indicated antibodies. C. Effects of LSDV122 on assembly of endogenous IFNAR complex and its downstream kinases. HEK293T cells (2 × 10^7^) were transfected with an empty vector or LSDV122-expressing plasmid for 24 hours. The cells were either left untreated or treated with IFN-β (200 ng/ml) for 30 minutes, then harvested for immunoprecipitation using control IgG, anti-IFNAR1 or anti-IFNAR2 antibodies. The lysates and immunoprecipitates were subjected to immunoblotting analysis with the indicated antibodies. These experiments were repeated at least twice with similar results.

IFN-I initially binds IFNAR2 with high affinity and subsequently recruits IFNAR1, forming an active receptor complex in which IFNAR1 associates with TYK2 and IFNAR2 engages JAK1 to trigger downstream signaling [11–13]. Since our earlier experiments suggest that LSDV122 interacts with IFNAR1 and IFNAR2, we next used flow cytometry to determine the effects of LSDV122 on binding of IFN-I to its receptor complex. The results indicated that overexpression of IFNAR2 but not LSDV122 or IFNAR1 increased binding of Flag-tagged IFN-α or IFN-β to HEK293T cells. Additionally, overexpression of LSDV122 or IFNAR1 did not affect IFN-α or IFN-β binding to IFNAR2 (Fig 5A). These results suggest that LSDV122 does not bind to IFN-I or interfere IFN-I binding to IFNAR2. These results are also consistent with previous reports that IFNAR2 binds IFN-β with higher affinity and forms a relatively stable binary complex, while IFNAR1 has lower binding affinity and is typically recruited to the preassembled IFNAR2-IFN-β complex to form the functional ternary receptor complex [29].

Since LSDV122 does not affect the binding of IFN-β to its receptor complex, we next investigated whether LSDV122 affects receptor complex formation and downstream kinase recruitments. Co-immunoprecipitation experiments showed that LSDV122 reduced the interactions between IFNAR1 and IFNAR2, IFNAR1 and TYK2, as well as IFNAR2 and JAK1 (Fig 5B). Consistently, overexpression of LSDV122 also reduced IFN-β-induced endogenous association of IFNAR1-IFNAR2, IFNAR1-TYK2 and IFNAR2-JAK1 (Fig 5C). These results suggest that LSDV122 associates with IFNAR1 and IFNAR2 and prevents formation of the receptor complex and recruitment of the downstream kinases JAK1 and TYK2, thereby impairing signal transduction and transcriptional induction of ISGs.

### LSDV122-deficiency enhances host antiviral response against LSDV

**Fig 6:**
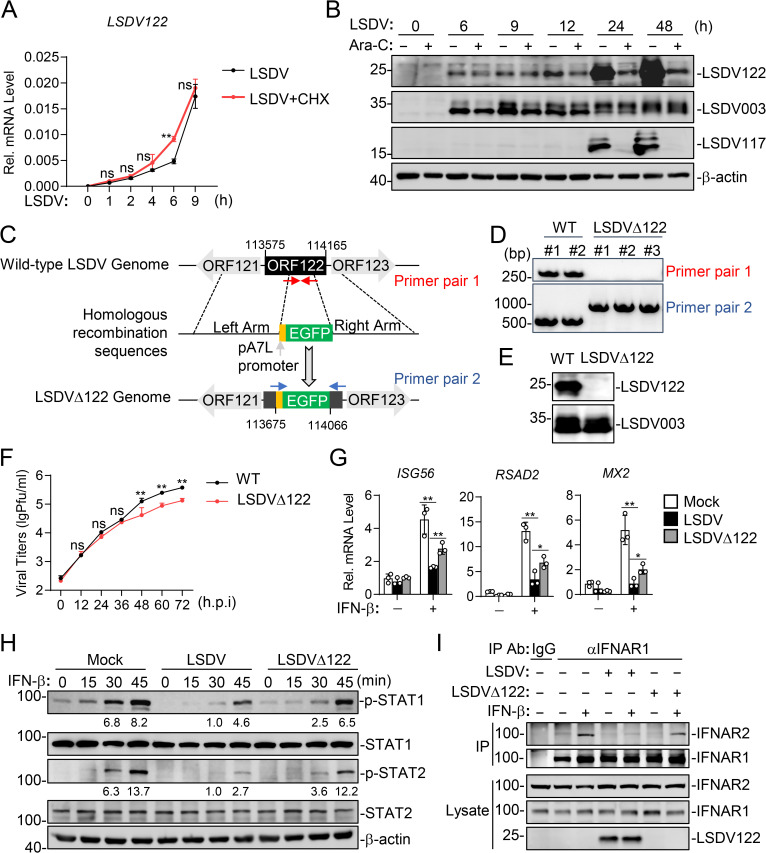
Deficiency of LSDV122 enhances IFN-β-induced antiviral response upon LSDV infection. A. Effects of cycloheximide (CHX) on transcription of *LSDV122* gene. MDBK cells (2 × 10^5^) were infected with LSDV (MOI = 1) for the indicated times in the absence or presence of CHX, followed by RT-qPCR analysis of mRNA level of the *LSDV122* gene. Data shown are mean ± SD (n = 3) from one representative experiment. B. Effects of cytosine arabinoside (Ara-C) on expression of LSDV122. MDBK cells (2 × 10^5^) were infected with LSDV (MOI = 1) for the indicated times in the absence or presence of Ara-C before immunoblotting analysis with a LSDV122 antibody. C. A schematic diagram for generation of LSDV strains with deletion of the *LSDV122* gene (LSDVΔ122). D. PCR verification of LSDVΔ122 purity. Viral DNA of wild-type LSDV or LSDVΔ122 (MOI = 2) was amplified with specific primers before gel electrophoresis analysis. E. Immunoblotting verification of LSDVΔ122 purity. MDBK cells (2 × 10^5^) were infected with wild-type LSDV or LSDVΔ122 (MOI = 2) for 24 hours. The cells were then lysed and expression of LSDV122 was detected by immunoblotting analysis. F.Growth kinetics of wild-type LSDV and LSDVΔ122. MDBK cells (2 × 10^5^) were infected with wild-type LSDV or LSDVΔ122 (MOI = 0.04) for the indicated times before plaque assays. Data shown are mean ± SD (n = 3) from one representative experiment. G. Effects of LSDV122-deficiency on IFN-β-induced transcription of antiviral genes. MDBK cells (2 × 10^5^) were left un-infected or infected with wild-type LSDV and LSDVΔ122 (MOI = 1) for 6 hours. The cells were then left untreated or treated with IFN-β (100 ng/ml) for 3 hours, followed by RT-qPCR analysis of mRNA levels of the indicated genes. Data shown are mean ± SD (n = 3) from one representative experiment. *P < 0.05; **P < 0.01 (unpaired t-test). H. Effects of LSDV122-deficiency on IFN-β-induced phosphorylation of STAT1 and STAT2. MDBK cells (2 × 10^5^) were left uninfected or infected with wild-type LSDV or LSDVΔ122 (MOI = 1) for 6 hours. The cells were then left untreated or treated with IFN-β (100 ng/ml) for the indicated times before immunoblotting analysis with the indicated antibodies. Phosphorylation levels were quantified by densitometry using ImageJ software, normalized to the corresponding total protein and β-actin. I. Effects of LSDV122-deficiency on assembly of endogenous IFNAR complex. MDBK cells (5 × 10^7^) were left uninfected or infected with wild-type LSDV or LSDVΔ122 (MOI = 2) for 18 hours. The cells were either left untreated or treated with IFN-β (500 ng/ml) for 30 minutes, then harvested for immunoprecipitation using control IgG or anti-IFNAR1 antibodies. The lysates and immunoprecipitates were subjected to immunoblotting analysis with the indicated antibodies. These experiments were repeated at least twice with similar results.

We next investigated the roles of LSDV122 in regulating IFN-I signaling and innate antiviral response upon LSDV infection. We firstly examined the expression kinetics of LSDV122 following LSDV infection. The protein synthesis inhibitor cycloheximide (CHX) did not impair the early transcription of LSDV122, indicating that its expression did not require de novo protein synthesis (Fig 6A). In addition, the DNA synthesis inhibitor cytosine arabinoside (Ara-C) had no marked effects on the early expression of LSDV122 or the annotated immediate-early gene LSDV003 at 6 and 9 hours post infection (h.p.i) [25], demonstrating that both genes were independent of viral DNA replication during the early stage (Fig 6B). However, at 24 and 48 h.p.i, Ara-C inhibited the expression of LSDV122 and the annotated late gene LSDV117 [25], while LSDV003 expression remained unaffected, indicating that LSDV122 also exhibits late-phase expression (Fig 6B). This temporal pattern is consistent with what has been reported for its VACV homolog A33R, which is expressed during both early and late stages of infection [30]. Together, these findings suggest that LSDV122 is expressed in both phases and should be classified as an early/late gene. We then generated a recombinant LSDV strain lacking the *LSDV122* gene (LSDVΔ122) from the highly pathogenic LSDV/China/Hainan/2021 strain via homologous recombination (Fig 6C). The genetic purity of LSDVΔ122 was confirmed by PCR analysis (Fig 6D), and the deficiency of LSDV122 expression was validated by immunoblotting analysis in cells infected with wild-type LSDV and LSDVΔ122 (Fig 6E). Replication kinetics assays showed that LSDVΔ122 exhibited a modest reduction in replication compared to the wild-type strain at 48, 60, and 72 h.p.i, suggesting that LSDV122 contributes to viral replication at late phase of infection (Fig 6F). Importantly, infection with LSDVΔ122 partially but significantly enhanced IFN-β-induced transcription of antiviral ISGs, including *ISG56*, *MX2*, and *RSAD2*, compared to wild-type LSDV (Fig 6G). IFN-β signaling was not completely restored, which may reflect the contribution of additional viral inhibitors such as LSDV135. Consistently, immunoblotting analysis indicated that infection of LSDVΔ122 resulted in higher STAT1 and STAT2 phosphorylation upon IFN-β stimulation than wild-type LSDV (Fig 6H). To further confirm that LSDV122 antagonizes IFN-I signaling by targeting the IFNAR complex during infection, we performed co-immunoprecipitation assays in MDBK cells infected with wild-type LSDV or LSDVΔ122. The results demonstrated that infection of wild-type LSDV but not LSDVΔ122 reduced IFN-β-induced endogenous association between IFNAR1 and IFNAR2 (Fig 6I). These findings suggest that LSDV122 disrupts IFNAR complex assembly to antagonize IFN-I signaling during LSDV infection.

**Fig 7:**
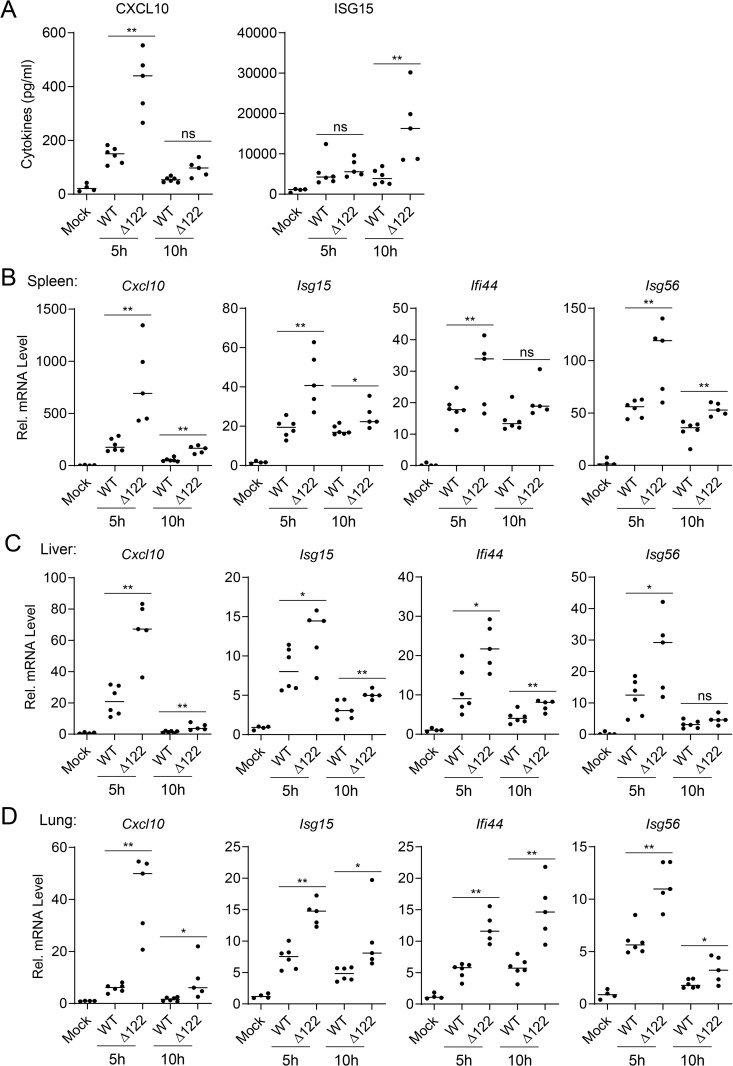
Deficiency of LSDV122 enhances host antiviral response *in vivo.* A. Effects of LSDV122-deficiency on LSDV-induced production of serum ISG15 and CXCL10. Seven-week-old C57BL/6 mice were intravenously injected with medium (mock, n = 4), LSDV (2.6 × 10^5^ pfu each mouse, n = 6) or LSDVΔ122 (2.6 × 10^5^ pfu each mouse, n = 5). The orbital blood of viral infected mice was collected at 5 and 10 hours post infection, and the orbital blood of mock injected mice was collected at 5 hours post injection for ELISA analysis of CXCL10 and ISG15 levels. B-D. Effects of LSDV122-deficiency on LSDV-induced transcription of antiviral genes in different tissues of mice. Seven-week-old C57BL/6 mice were intravenously injected with medium (mock, n = 4), LSDV (2.6 × 10^5^ pfu each mouse, n = 6) or LSDVΔ122 (2.6 × 10^5^ pfu each mouse, n = 5). Spleen (B), liver (C) and lung (D) of mice were collected at 5 or 10 hours post viral infection or 5 hours post mock injection for RT-qPCR analysis of mRNA levels of the indicated genes. Data shown in A-D are mean ± SD. These experiments were repeated at least twice with similar results. *P < 0.05; **P < 0.01 (unpaired t-test).

We further evaluated the roles of LSDV122 in regulating innate antiviral immune response *in vivo*. C57BL/6 mice were intraperitoneally infected with either wild-type LSDV or LSDVΔ122. ELISA analysis showed that the levels of ISG15 and CXCL10 in the sera were significantly increased in mice infected with LSDVΔ122 compared to those infected with wild-type LSDV (Fig 7A). In addition, RT-qPCR analysis of tissue samples revealed that deletion of LSDV122 markedly enhanced the transcription of multiple antiviral genes, including *Isg56*, *Isg15*, *Ifi44*, and *Cxcl10*, in the spleens, livers and lungs of mice (Fig 7B–7D). Collectively, these results suggest that deficiency of LSDV122 enhances host antiviral response upon LSDV infection.

## Discussion

IFN-I activates the JAK-STAT signaling to induce antiviral ISGs, which collectively mediate antiviral defense, antiproliferative activity, and adaptive immune stimulation [16,17]. LSDV has evolved multiple immune escape strategies to inhibit IFN-I signaling and promote viral replication, but the underlying mechanisms are not fully understood. Therefore, investigating how LSDV antagonizes IFN-I-triggered antiviral defense is essential for understanding viral immune escape and developing improved strategies for LSD control.

Our findings demonstrate that LSDV infection markedly suppresses IFN-β-induced phosphorylation of STAT1 and STAT2, as well as the transcription of downstream ISGs. These observations suggest that LSDV may encode viral antagonists of host innate immune response to evade IFN-β-triggered antiviral response. Through a systematic screen of LSDV proteins, we identified LSDV122 as a novel antagonist of IFN-β signaling. Ectopic expression of LSDV122 inhibited IFN-β-triggered phosphorylation of STAT1 and STAT2, as well as the transcription of multiple ISGs in human HEK293T and bovine MDBK cells. In contrast, LSDV122 had no marked effects on IFN-γ-induced phosphorylation of STAT1 or transcription of its downstream genes. These results suggest that LSDV122 specifically antagonizes IFN-I- but not IFN-II-induced JAK-STAT signaling.

Mechanistically, we found that LSDV122 was localized to the cell membrane and interacted with IFNAR1 and IFNAR2. Domain mapping experiments demonstrated that the transmembrane domain of LSDV122 is sufficient to interact with IFNAR1 and IFNAR2, while LSDV122 mutant containing the transmembrane domain and cytoplasmic fragment has increased ability to interact with IFNAR1 and IFNAR2. On the other hand, the IFNAR1 and IFNAR2 mutants containing their respective transmembrane domain and cytoplasmic fragment, but not the transmembrane domain alone, or the transmembrane domain plus the extracellular domain, are sufficient to interact with LSDV122. These results suggest that the stable interaction of LSDV122 with IFNAR is dependent on the cooperative engagement of their respective transmembrane and cytoplasmic regions. While the transmembrane domains may provide membrane anchoring and hydrophobic contacts, the cytoplasmic domains likely contribute specific residues or structural motifs that facilitate complex formation. Despite LSDV122 specifically engages IFNAR, its overexpression does not affect IFN-I binding to IFNAR, and LSDV122 itself does not bind to IFN-I. Rather, LSDV122 impairs the assembly of the IFNAR complex and reduces recruitment of the downstream kinases JAK1 and TYK2 to the receptor complex, ultimately inhibiting downstream JAK/STAT signaling and ISGs induction.

Through a systematic characterization of the expression kinetics and functional properties of LSDV122, our results showed that *LSDV122* is an early/late gene, and its deletion reduced viral replication at late phase of infection. Importantly, infection with LSDV122-deficient virus (LSDVΔ122) enhanced IFN-β-induced phosphorylation of STAT1 and STAT2, as well as transcription of downstream antiviral ISGs compared to wild-type LSDV. These results suggest that LSDV122 exhibits dual functions, including suppression of IFN-I signaling and promotion of late-stage viral replication. Consistent with the results *in vitro*, LSDV122-deficiency increased the levels of ISG15 and CXCL10 in the sera of virus-infected mice and enhanced the transcription of multiple antiviral genes in the spleens, livers, and lungs. Thus, deficiency of LSDV122 enhances IFN-I response both *in vitro* and *in vivo*, indicating its role in attenuating innate antiviral response upon LSDV infection.

We also evaluated the functions of LSDV122 homologs derived from related poxviruses. Homologous proteins from closely related Capripoxviruses, including SPPV and GTPV, share over 95% sequence identity with LSDV122 and similarly antagonized IFN-β-induced transcription of *ISG* genes. In contrast, homologs from more distantly related poxviruses, such as VACV, MPXV, and MYXV, had no marked effects on the expression of these genes, which likely reflects limited amino acid sequence conservation or host-specific differences. Furthermore, we identified LSDV135, a homolog of VACV B18R that functions as a soluble IFN-β receptor [19,25], which also plays a critical role in suppressing IFN-β response. This likely explains why deletion of LSDV122 alone does not fully restore IFN-β-induced innate antiviral signaling. Notably, LSDV122 and LSDV135 exhibit distinct mechanisms of action and subcellular localization, suggesting that they may function in a complementary manner to efficiently inhibit IFN-I signaling during LSDV infection.

**Fig 8:**
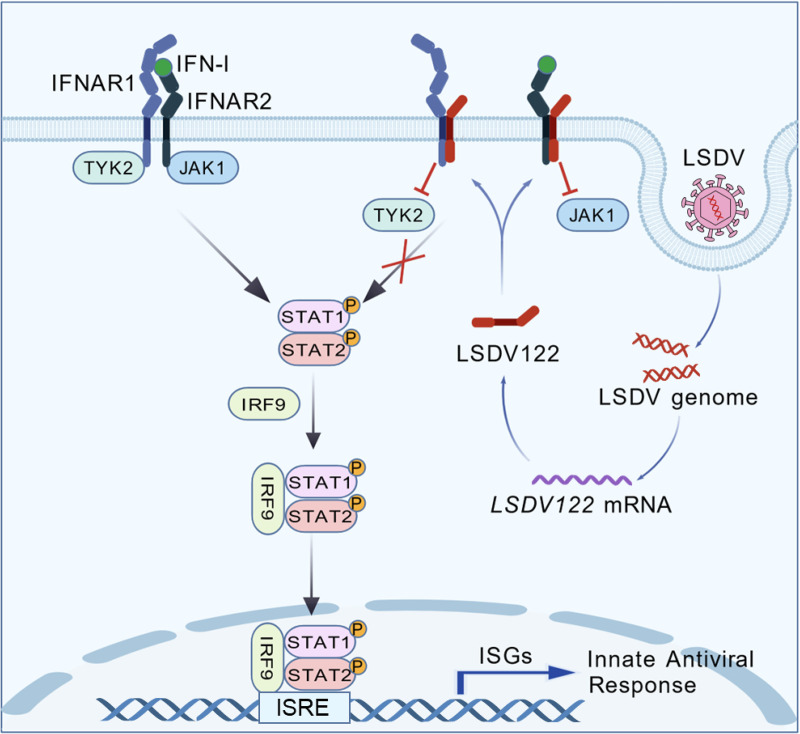
A model of antagonization of IFN-I signaling by LSDV122. Following LSDV infection, the viral protein LSDV122 interacts with both subunits of the IFN-I receptor, IFNAR1 and IFNAR2, via their respective transmembrane and intracellular domains. This impairs the assembly of the IFNAR complex and recruitment of downstream kinases JAK1 and TYK2 to the complex, leading to suppression of IFN-I-triggered induction of ISGs and antiviral response.

In conclusion, our study identifies LSDV122 as an antagonist of IFN-I signaling by targeting the IFNAR complex and preventing the recruitment of JAK1 and TYK2 (Fig 8). These findings provide mechanistic insights into how LSDV subverts host antiviral immunity and suggest that LSDV122 is a promising target for rational design of live-attenuated LSDV vaccines.

## Materials and methods

### Ethics statement

All animals were handled strictly with good animal practices according to the Animal Ethics Procedures and Guidelines of the People’s Republic of China. The study was approved by the Animal Ethics Committee of the Lanzhou Veterinary Research Institute of the Chinese Academy of Agricultural Sciences (approval number: LVRIAEC-2025–015).

### Reagents, antibodies, cells and viruses

Trypsin (C100C1, NCM Biotech), fetal bovine serum (SA211.02, Cellmax), penicillin and streptomycin (PB180120, Pricella), Dulbecco’s Modified Eagle Medium (C11965500BT, Gibco), puromycin (AMR-J593, VWR), protein A + G agarose (P2055, Beyotime), PMSF (P7626, Sigma), recombinant human IFN-β (300–02BC, ThermoFisher Scientific), recombinant human IFN-γ (300–02, ThermoFisher Scientific), SYBR Green Supermix (Q312, Vazyme), HiScript II Select RT SuperMix for qPCR (R323, Vazyme), and aprotin, leupeptin, β-glycerophosphate disodium salt, Ara-C, CHX and sodium orthovanadate (HY-P0017, HY-18234A, HY-126304, HY-13605, HY-12320 and HY-D0852 respectively, MCE) were purchased from the specified manufacturers.

Mouse monoclonal antibodies against HA (66006, Proteintech), Rabbit monoclonal antibodies against HA (H6908, Sigma), Mouse monoclonal antibodies against Flag (AP0530, Sigma), HRP-Flag (ZB15939, Servicebio), IgG (I5381 and I5006, Sigma), p-JAK1 (3331S, Cell Signaling Technology), JAK1 (A25841, ABclonal), p-TYK2 and TYK2 (9321S and 14193S, Cell Signaling Technology), p-STAT1 (9167S, Cell Signaling Technology), STAT1 (sc-417, Santa Cruz Biotechnology), p-STAT2 and STAT2 (88410S and 72604S, Cell Signaling Technology), IFNAR1 (A0575, ABclonal), IFNAR2 (53883s, Cell Signaling Technology), PE anti-DYKDDDDK Tag (Biolegend, 637310), Anti-mouse IgG (H + L), F(ab’)2 Fragment (Alexa Fluor 594 Conjugate) (8890, Cell Signaling Technology) and Alexa Fluor 488 goat anti-rabbit IgG (H + L) (A11008, Invitrogen) were purchased from the specified manufacturers. LSDV117 antibody was provided by professor Jun-Zheng Du (Lanzhou Veterinary Research Institute, Chinese Academy of Agricultural Sciences).

HEK293T and HeLa cells were obtained from the American Type Culture Collection (ATCC). MDBK cells were purchased from the China Center for Type Culture Collection (CCTCC).

The LSDV/China/Hainan/2021 strain (GenBank accession: PV796583) used in this study has been previously described [31].

### Plasmids

The LSDV protein expression clones were synthesized by GenScript. Expression plasmids for HA-tagged LSDV122, IFNAR1, IFNAR2, and their truncation mutants, as well as Flag-tagged IFNAR1, IFNAR2, JAK1, TYK2, were constructed using standard molecular biology techniques.

### Preparation of LSDV122 antibody

The prokaryotic expression vector pET-30c encoding an LSDV122 fusion construct comprising amino acids 1–44 and 72–196 was transformed into *E. coli* BL21 (DE3) competent cells. A 10 mL LB starter culture was inoculated from a single colony and grown overnight at 37°C with shaking at 220 rpm. The culture was then diluted 1:50 into 500 mL LB medium and incubated until mid-log phase (OD_600_ = 0.6-0.8). Protein expression was induced with 0.5 mM IPTG at 16°C for 10 hours. The recombinant protein was purified under denaturing conditions following inclusion body isolation protocols. The purified protein was subsequently used as an antigen to immunize 8-week-old BALB/c mice via multi-site subcutaneous injections. Mice received five immunizations at 14-day intervals, and polyclonal antibodies were collected from serum samples one week after the final immunization.

### Generation of stable cell lines

A cDNA corresponding to LSDV122-coding sequence was cloned into the pHAGE vector, which was co-transfected with packaging plasmids psPAX2 and pCMV-VSV-G into HEK293T cells. Forty-eight hours after transfection, the viruses were harvested and used to infect MDBK cells. The infected cells were selected with puromycin (1.5 μg/mL) for 6 days to establish stable cell lines.

### Generation of LSDVΔ122

LSDVΔ122 was generated by homologous recombination in MDBK cells. The recombinant plasmid was firstly constructed using pUC18 as the backbone. The plasmid contained left and right homology arms flanking the *LSDV122* locus, as well as the *EGFP* reporter gene driven by the LSDV pA7L promoter, which was inserted into the coding region of *LSDV122*. MDBK cells were infected with wild-type LSDV (LSDV/China/Hainan/2021 strain) for 12 hours and then transfected with the homologous recombinant plasmid using Fugene transfection reagent. After 3 days, cells were frozen-thawed and seeded onto MDBK cells in 96-well plates by limiting dilution assay. After several rounds of dilution screening and amplification, the purified LSDVΔ122 was obtained. The purity of LSDVΔ122 was determined by PCR and immunoblotting analysis. The whole genome of LSDVΔ122 was sequenced, which confirmed the targeted insertion of the EGFP reporter cassette and lack of other unintended mutations in the genome.

The sequences of the PCR primers were as follows:

Primer pair 1: 5’-GATATTCCAAAGAGTGGAAC-3’ and

5’-ATAGCTTCTCATCTCATCGC-3’;

Primer pair 2: 5’-GATATTCCAAAGAGTGGAAC-3’ and

5’-ACGCTGAACTTGTGGCCGTT-3’.

### RT-qPCR

Total RNA was isolated, and cDNA was synthesized by reverse transcription for qPCR analysis. Relative mRNA levels were calculated using GAPDH as the internal control. For most experiments, the 2^-ΔΔCt method was used with untreated cells as the calibrator (expression in control cells = 1). For viral gene measurements in Fig 6A, where untreated cells have no detectable transcripts, the 2^-ΔCt method was applied. The sequences of the qPCR primers were as follows:

Human *GAPDH*: 5’-GACAAGCTTCCCGTTCTCAG-3’ and

5’-GAGTCAACGGATTTGGTCGT-3’;

Human *ISG56*: 5’-TCATCAGGTCAAGGATAGTC-3’ and

5’-CCACACTGTATTTGGTGTCTAGG-3’;

Human *IRF1*: 5’-AGAGCAAGGCCAAGAGGAAGTCAT-3’ and

5’-AAGTCCTGCATGTAGCCTGGAACT-3’;

Human *GBP1*: 5’-TAGCAGACTTCTGTTCCTACATCT-3’ and

5’-CCACTGCTGATGGCATTGACGT-3’;

Human *RSAD2*: 5’-CCAGTGCAACTACAAATGCGGC-3’ and

5’-CGGTCTTGAAGAAATGGCTCTCC-3’;

Human *IFI44*: 5’-GTGAGGTCTGTTTTCCAAGGGC-3’ and

5’-CGGCAGGTATTTGCCATCTTTCC-3’;

Bovine *GAPDH*: 5’-AGGTCGGAGTGAACGGATTC-3’ and

5’-ATGGCGACGATGTCCACTTT-3’;

Bovine *ISG56*: 5’-TCACAGCAACCATGAGTTATAAAG-3’ and

5’-ATCTCCTCCAAGACCCTGTT-3’;

Bovine *IRF1*: 5’-CAGACAAGCGTGGATGGGAA-3’ and

5’-CAGGCCAATATAACCCCCAGG-3’;

Bovine *GBP1*: 5’-TGTGAGCTTCTTTCCAGACTTTG-3’ and

5’-CTTTTGGGCTGGTACCTTTCT-3’;

Bovine *RSAD2*: 5’-CTGGAGGAGGCCAAGAAAGG-3’ and

5’-CCGTTGCTGACAATGCTGAC-3’;

Bovine *IFI44*: 5’-ACAGTCTGCCCATTGCTGAA-3’ and

5’-CCACCATCTCATGGGAGAGC-3’;

Bovine *MX2*: 5’-CTACCGCAACATTACGCAGC-3’ and

5’-TCAGATCTGGGACCTCAGGG-3’;

Mouse *Gapdh*: 5’-ACGGCCGCATCTTCTTGTGCA-3’ and

5’-ACGGCCAAATCCGTTCACACC-3’;

Mouse *Isg15*: 5’-CATCCTGGTGAGGAACGAAAGG-3’ and

5’-CTCAGCCAGAACTGGTCTTCGT-3’;

Mouse *Isg56*: 5’-ACAGCAACCATGGGAGAGAATGCTG-3’ and

5’-ACGTAGGCCAGGAGGTTGTGCAT-3’;

Mouse *Cxcl10*: 5’-ATCATCCCTGCGAGCCTATCCT-3’ and

5’-GACCTTTTTTGGCTAAACGCTTTC-3’;

Mouse *Ifi44*: 5’-ATGCACTCTTCTGAGCTGGTGG-3’ and

5’-TCAGATCCAGGCTATCCACGTG-3’;

*LSDV122*: 5’-GCATTCGCAGGTTCCACTAT-3’ and

5’-ACGAAACCATTGATGCCATA-3’.

### Confocal microscopy

HeLa cells or MDBK cells were seeded on coverslips in 24-well plates. After 24 hours, HeLa cells were transfected with the indicated plasmids for 20 hours and MDBK cells were infected with LSDV for 18 hours. The cells were fixed with 4% paraformaldehyde for 30 min and then permeabilized with 0.1% Triton X-100 for 15 min. Subsequently, cells were blocked in 1% BSA and stained with the indicated primary and secondary antibodies. Imaging of cells was carried out using a Zeiss confocal microscope.

### Coimmunoprecipitation and immunoblotting analysis

Cells were lysed in lysis buffer (20 mM Tris-HCl pH 7.4, 1% NP-40, 150 mM NaCl, 1 mM EDTA and protease inhibitors) at 4°C for 10 min, followed by sonication. The lysates were centrifuged at 13,000 rpm for 10 min at 4°C, and the resulting supernatants were incubated with the indicated antibodies for immunoprecipitation. The antibody-protein complexes were captured with protein A/G beads and then washed three times with high-salt washing buffer (750 mM NaCl, 50 mM Tris-HCl, pH 7.4). The bound proteins were separated by SDS-PAGE and analyzed by immunoblotting with the indicated antibodies.

### Viral plaque assay

MDBK cells were infected with wild-type LSDV or LSDVΔ122 (MOI = 0.01) for the indicated times. Both cells and supernatants were harvested and freeze-thawed to obtain viral suspensions. Serial dilutions of these suspensions were used to infect MDBK cells for 2 hours at 37°C. The cells were then overlaid with 1.5% methylcellulose and incubated for 96 hours before plaque counting.

### Animal experiments

Six-month-old Holstein cattle were purchased from Gansu Qianjin Animal Husbandry Technology Co., Ltd. Cattle were intravenously injected with wild-type LSDV (2 × 10^6^ pfu). At 14 days post-infection, the lesion and non-lesion skin samples from the same cattle were dissected for RT-qPCR experiments.

Six-week-old female C57BL/6 mice were purchased from Lanzhou Veterinary Research Institute of the Chinese Academy of Agricultural Sciences. Mice were intravenously injected with wild-type LSDV or LSDVΔ122 at a dose of 2.6 × 10^5^ pfu per mouse. At 5 and 10 hours post-infection, orbital blood samples were collected, and the levels of Isg15 and Cxcl10 in the serum were measured using ELISA kits (CSB-EL011843MO, CUSABIO; EK0736, BOSTER). Spleens, livers and lungs were harvested and homogenized using steel beads (Qiagen) in a Tissuelyser for 5 min. Total RNA was then extracted and subjected to RT-qPCR analysis.

### Facility biosafety statement

All experiments with live LSDV were conducted in the National Foot and Mouth Disease Reference Laboratory (ABSL-3) at the Lanzhou Veterinary Research Institute of the Chinese Academy of Agricultural Sciences. The experiments were approved by the Ministry of Agriculture and Rural Affairs (approval number: 07140020250302) and the China National Accreditation Service for Conformity Assessment (approval number: CNAS BL0098).

### Statistics analysis

Unpaired Student’s *t* test was used for statistical analysis with GraphPad Prism Software. The number of asterisks represents the degree of significance with respect to P values. Statistical significance was set at P < 0.05, * or P < 0.01, **.

## Supporting information

S1 Raw Data Excel file containing the numerical values used for statistical analyses and for generating the figures in this study. (XLSX)
